# Alterations in regional vascular geometry produced by theoretical stent implantation influence distributions of wall shear stress: analysis of a curved coronary artery using 3D computational fluid dynamics modeling

**DOI:** 10.1186/1475-925X-5-40

**Published:** 2006-06-16

**Authors:** John F LaDisa, Lars E Olson, Hettrick A Douglas, David C Warltier, Judy R Kersten, Paul S Pagel

**Affiliations:** 1 Department of Pediatrics (Division of Cardiology), Stanford University, Stanford, California, USA; 2 Department of Anesthesiology, the Medical College of Wisconsin and the Clement J. Zablocki Veterans Affairs Medical Center, Milwaukee, Wisconsin, USA; 3 Department of Medicine (Division of Cardiovascular Diseases), the Medical College of Wisconsin and the Clement J. Zablocki Veterans Affairs Medical Center, Milwaukee, Wisconsin, USA; 4 Department of Pharmacology and Toxicology, the Medical College of Wisconsin and the Clement J. Zablocki Veterans Affairs Medical Center, Milwaukee, Wisconsin, USA; 5 Department of Biomedical Engineering, Marquette University, Milwaukee, Wisconsin, USA

## Abstract

**Background:**

The success of stent implantation in the restoration of blood flow through areas of vascular narrowing is limited by restenosis. Several recent studies have suggested that the local geometric environment created by a deployed stent may influence regional blood flow characteristics and alter distributions of wall shear stress (WSS) after implantation, thereby rendering specific areas of the vessel wall more susceptible to neointimal hyperplasia and restenosis. Stents are most frequently implanted in curved vessels such as the coronary arteries, but most computational studies examining blood flow patterns through stented vessels conducted to date use linear, cylindrical geometric models. It appears highly probable that restenosis occurring after stent implantation in curved arteries also occurs as a consequence of changes in fluid dynamics that are established immediately after stent implantation.

**Methods:**

In the current investigation, we tested the hypothesis that acute changes in stent-induced regional geometry influence distributions of WSS using 3D coronary artery CFD models implanted with stents that either conformed to or caused straightening of the primary curvature of the left anterior descending coronary artery. WSS obtained at several intervals during the cardiac cycle, time averaged WSS, and WSS gradients were calculated using conventional techniques.

**Results:**

Implantation of a stent that causes straightening, rather than conforms to the natural curvature of the artery causes a reduction in the radius of curvature and subsequent increase in the Dean number within the stented region. This straightening leads to modest skewing of the velocity profile at the inlet and outlet of the stented region where alterations in indices of WSS are most pronounced. For example, time-averaged WSS in the proximal portion of the stent ranged from 8.91 to 11.7 dynes/cm^2 ^along the pericardial luminal surface and 4.26 to 4.88 dynes/cm^2 ^along the myocardial luminal surface of curved coronary arteries as compared to 8.31 dynes/cm^2 ^observed throughout the stented region of a straight vessel implanted with an equivalent stent.

**Conclusion:**

The current results predicting large spatial and temporal variations in WSS at specific locations in curved arterial 3D CFD simulations are consistent with clinically observed sites of restenosis. If the findings of this idealized study translate to the clinical situation, the regional geometry established immediately after stent implantation may predispose portions of the stented vessel to a higher risk of neointimal hyperplasia and subsequent restenosis.

## Background

There is a putative link between altered vascular geometry and locations of neointimal hyperplasia (NH) and cellular proliferation *in vivo*[1-3]. Stent implantation effectively restores vascular patency distal to a stenosis, but changes in regional geometry created by the implanted stent may also affect local hemodynamics in a manner conducive to the development of NH and subsequent restenosis[4-6]. We recently demonstrated that distributions of low wall shear stress (WSS) that occur after stent implantation correlate with the development of NH in rabbit iliac arteries *in vivo*[7]. As this NH develops, the geometry and associated distributions of WSS are temporally altered in a manner that progressively abolishes WSS disparity within the stented region. We have further demonstrated using 3D computational fluid dynamics (CFD) modeling that the geometric properties of an implanted stent (e.g., number, width and thickness of stent struts), the severity of stent foreshortening, and local scaffolding created by the stent affect indices of WSS associated with this process[8-10].

Our previous 3D CFD modeling investigations were conducted using simplified representations of normal coronary arteries modeled as cylindrical tubes. In reality, the coronary arteries follow the curvature of the heart and it is highly likely that straightening of this natural curvature will affect indices of WSS within the stented region. A variety of stent designs are currently available and used clinically, each with unique geometric and mechanical properties. Stent flexibility is especially desirable, as a more flexible stent may increase the probability of successful deployment in tortuous distal lesions with various degrees of calcification and atherosclerosis. However, despite the flexibility of available stents, Wentzel *et al*. demonstrated that implantation of a stent may cause straightening of the coronary artery segment where the stent is deployed[11]. This action may have an important acute or chronic impact on distributions of WSS in the proximal and distal regions of the stent and may establish adverse indices of WSS that influence the long-term pattern of NH. Thus, we tested the hypothesis that stent-induced alterations in regional vascular geometry influence the distribution of indices of WSS using 3D CFD models of theoretical implanted stents that conform to or straighten the primary coronary arterial curvature quantified *in vivo*.

## Methods

### Construction of stented computational vessels

**Figure 1:**
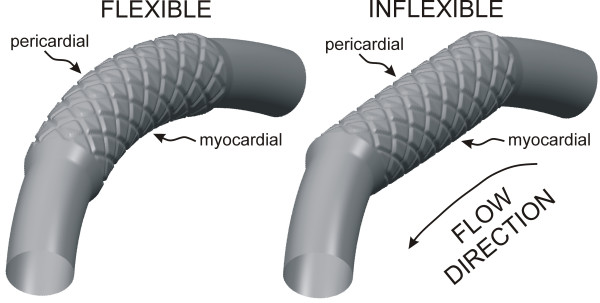
Computational vessels implanted with 12 mm stents that conform to (flexible, left) or cause straightening of (inflexible, right) an ideal, curved coronary artery constructed based on the diameter and curvature measured in a canine left anterior descending coronary artery *in vivo*. The pericardial and myocardial luminal surfaces are indicated on the figure. Computational vessels were created consisting of 8 axial and circumferential repeating strut sections that were implanted using a stent-to-artery diameter ratio of 1.2 The thickness of all stent struts was 0.096 mm. The width of all stent struts was 0.197 mm.

A custom automated geometric construction and mesh generation algorithm was used to create idealized computational arteries containing a slotted-tube stent embedded within a normal computational vessel based on blood flow and diameter measurements obtained from canine left anterior descending coronary arteries (LAD)[12]. The algorithm allows for the alteration of several geometric parameters including the number, width and thickness of stent struts[10], resulting scaffolding created by the stent[9], vascular and stent diameters (i.e. deployment ratio)[10], deployed stent length[8], and curvature. All computational vessels were composed of structured hexahedral control volumes arranged in a four-domain butterfly design that exploited symmetric stent and vessel characteristics to model half of the computational vessel. Computational vessels were created consisting of 8 axial and circumferential repeating strut sections that were implanted using a stent-to-artery diameter ratio of 1.2 (Fig 1)[4,13]. The diameter of the unstented portions of the computational vessels for all simulations was 2.74 mm. The computational vessel within the stented region conformed to the geometry of the implanted stent[4,5,14,15]. The length of the computational stents was 12 mm. The thickness of all stent struts modeled in the current investigation was 0.096 mm. The width of all stent struts was 0.197 mm. The ability of the stent to influence distributions of WSS was investigated by creating curved arteries that conform to an average radius of curvature of 20.3 mm obtained from measurements in a canine left anterior descending coronary artery *in vivo*[12], or cause straightening of the stented segment of the artery[11](Fig 1). The simulations presented in the current investigation are differentiated as "inflexible" or "flexible", but this convention is used to merely describe the resulting geometry of the vessels that is caused by implantation of stents having these respective different mechanical properties and is not meant to imply that one stent was modeled as rigid while the other was deformable.

### Computational model simulations

**Figure 2:**
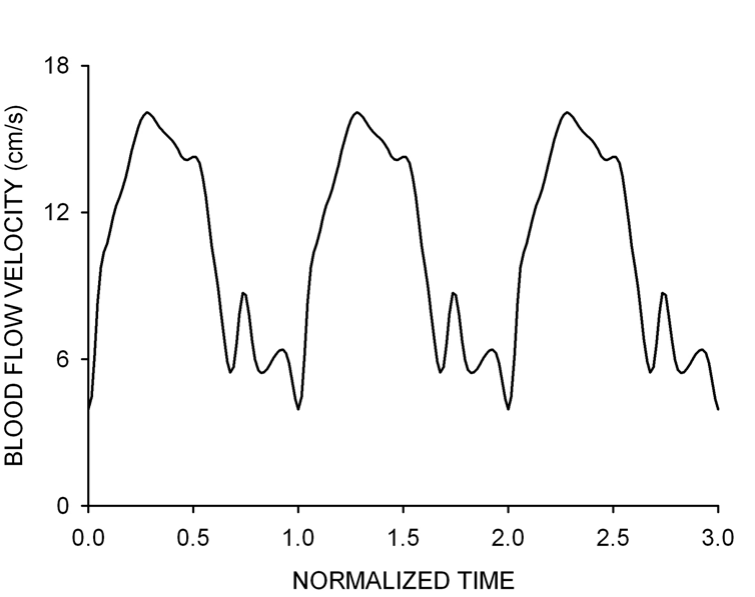
A representative waveform depicting the average blood flow velocity measured in the proximal portion of a canine left anterior descending coronary artery during a representative cardiac cycle. This waveform was used for the time-dependent simulations conducted in the current investigation.

Time-dependent simulations were performed using the commercially available software package CFD-ACE (CFDRC; Huntsville, AL, ). This software uses a finite volume approach to solve the Navier-Stokes equations at the center of each hexahedral control volume. Theoretical arteries were subjected to a blood flow velocity waveform obtained from a normal canine coronary artery under resting conditions (Fig 2). A plug flow velocity profile was imposed at the inlet of each vessel and additional length was added to all arteries to allow for fully developed flow[10,16]. The flow domain was initialized with an axial velocity of 10.5 cm/s at the start of each simulation to increase the likelihood of convergence. A zero pressure boundary condition was imposed at the outlet of the computational vessels. Simulations were conducted using a backward Euler temporal differencing method to investigate time-dependent changes in indices of WSS within each computational vessel.

Computational simulations were conducted assuming a Newtonian, incompressible fluid with a density of 1.06 g/cm^3 ^and viscosity of 3.7 cP[17,18]. The average Reynolds, Dean[19] and Womersley numbers during the cardiac cycle were approximately 105, 27.3 and 2.91, respectively. These values are similar to those reported in other studies examining fluid flow through curved sections with similar dimensions[19,20]. Simulations were allowed to converge for a minimum of 400 iterations or a 10–4 reduction in the solution residuals per time step.

### Calculation of indices of wall shear stress

Wall shear stress was determined as the product of viscosity and shear rate. A detailed discussion of this calculation is presented elsewhere[9]. Briefly, the CFD-ACE flow solver calculates shear rate during incompressible flow using the second invariant of the strain rate tensor. Thus, shear rate () was determined as



where *u*, *v*, and *w *are the *x*, *y *and *z *components of velocity vector, **u**, respectively.

This definition accounts for pure shear as well as extensional or elongational deformation in the flow domain.

Spatial wall shear stress gradients (WSSG) were calculated during post-processing as discussed previously[10,21]. WSSG was used to quantify the influence of non-uniform WSS on adjacent intravascular cells. Previous studies have suggested that this spatial WSS inhomogeneity may correlate with the location of neointimal hyperplasia [22-24] as was recently observed following chronic stent implantation into rabbit iliac arteries[7]. The axial and circumferential components of the WSS vector were most likely to cause expansion of intracellular gaps and disrupt intracellular junctions[25]. As a result, WSSG was calculated as , where τ_*w *_is the WSS in the axial (*z*) and circumferential (θ) directions, respectively. WSS and WSSG observed overlying stent struts were ignored in the current analysis because these areas do not contain biologically active tissue immediately after acute stent implantation.

### Quantification of simulation results

The threshold for comparing distributions of low WSS between simulations was 5 dynes/cm^2 ^for comparison to previous work[9,10] and because vascular regions subjected to WSS below this value have been shown to strongly correlate with sites of intimal thickening[1,26]. Temporal distributions of WSS were obtained in the center of the first proximal and last distal axial strut diamond of the stent along both the pericardial (i.e outer) and myocardial (i.e. inner) luminal surfaces. Time-averaged WSS (TAWSS) was then calculated in each of these regions to elucidate the influence of curvature on the temporal potential for the development of neointimal hyperplasia since low TAWSS is thought to be associated with regions of cellular proliferation[21].

WSSG have also been used previously to examine the hypothesis that normally confluent cells react to nonuniform distributions of WSS in a way that promotes NH[23,27,28]. The percentage of the vessel wall subjected to WSSG values above 20 dynes/cm^3 ^was quantified and compared between simulations in the current investigation. WSSG of this order of magnitude previously correlated with areas of NH in the toe region of an end-to-side arterial anastomosis [28-30] and in rabbit iliac arteries after stent implantation[7].

### Mesh and time-step independence

Simulations were performed on two Dell Optiplex GX270 2.4 GHz workstations each with 2 Gbyte of RAM allowing for simulation convergence at a rate of approximately 25 time-steps per day. Several simulations were performed to investigate spatial mesh independence. The mesh density was four times greater in stented as compared to unstented regions of the computational vessels resulting in over 500,000 nodes per half of each computational artery. Results were considered spatially independent of the computational mesh when the disparity between distributions of WSS between successive mesh densities was less than 6% [17,31]. Time-step independence was examined by subjecting computational vessels to the coronary artery blood flow velocity waveform illustrated in Fig 2 using time-step increments of 10.9, 8.0 or 5.4 ms. Three consecutive cardiac cycles with a period of 0.57 seconds each were stipulated for simulation convergence in the event that periodic WSS values did not ensue after the first or second cardiac cycles, and distributions of WSS were compared at equivalent points during each cardiac cycle and between waveform permutations. The results demonstrated that a single cardiac cycle was sufficient to allow for the evolution of initial conditions, and simulation results became periodic shortly after the first cardiac cycle. A time step increment of 8.0 ms was sufficient to resolve temporal distributions of WSS within the stented and unstented regions of each vessel.

## Results

Indices of WSS corresponding to peak and mean blood flow velocity during deceleration are shown in Table 1. Lower WSS was observed within the proximal transition region leading into the stent and within the stented region of all simulations. Stagnation zones were observed around stent struts. The total area of the stented region exposed to WSS < 5 dynes/cm^2 ^at the point of the cardiac cycle corresponding to peak, but not mean, blood flow velocity was greater for the flexible as compared to the inflexible stent (29.0 vs 24.4 mm^2^, respectively). The total area of the computational vessel subjected to WSSG greater than 20 dynes/cm^3 ^was also greater in the curved stented section as compared to the straightened stented section during peak and mean blood flow. In contrast, the maximum WSSG observed throughout the stented region during peak blood flow was greater in inflexible as compared to flexible theoretical stents (2470 vs 2210 dynes/cm^3^; Table 1).

**Table 1:** Indices of wall shear stress

	**FLEXIBLE**		**INFLEXIBLE**	
**Simulation**	**Peak**	**Mean**	**Peak**	**Mean**
**Total area exposed to WSS < 5 dynes/cm^2 ^(mm^2^)**	29.0	99.1	24.4	103.7
**Total area exposed to WSSG > 20 dynes/cm^3 ^(mm^2^)**	91.7	73.8	78.9	68.3
**WSS_min _(dynes/cm^2^)**	0.56	0.07	0.60	0.05
**WSS_max _(dynes/cm^2^)**	45.3	21.7	53.0	25.5
**WSSG_max _(dynes/cm^3^)**	2210	1080	2470	1030

Abbreviations: WSS = wall shear stress; WSSG = wall shear stress gradient

**Figure 3:**
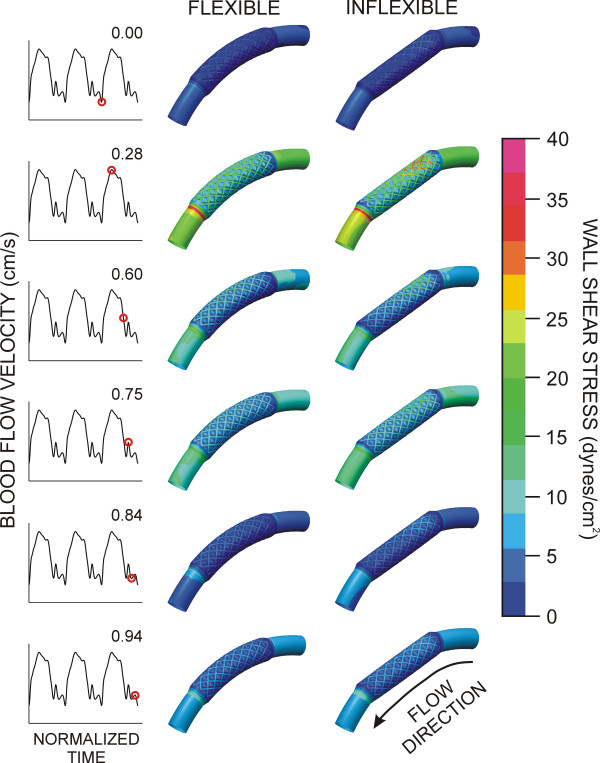
Time-dependent alterations in spatial wall shear stress throughout the cardiac cycle in computational vessels implanted with 12 mm stents that conform to (flexible, left) or cause straightening of (inflexible, right) an idealized and curved coronary artery.

**Figure 4:**
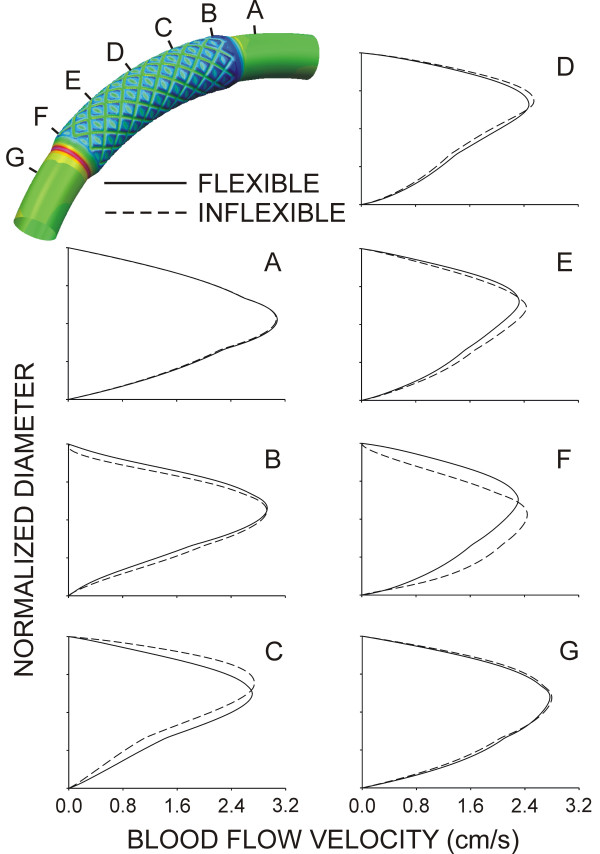
Centerline blood flow velocity profiles along the radial plane of symmetry at several axial locations of computational vessels implanted with 12 mm stents that conform to (flexible, solid lines) or cause straightening of (inflexible, dashed lines) an ideal, curved coronary artery. For reference, the pericardial luminal surface would be located closest to the top of each profile while the myocardial luminal surface would be located nearest the abscissa of each panel.

**Figure 5:**
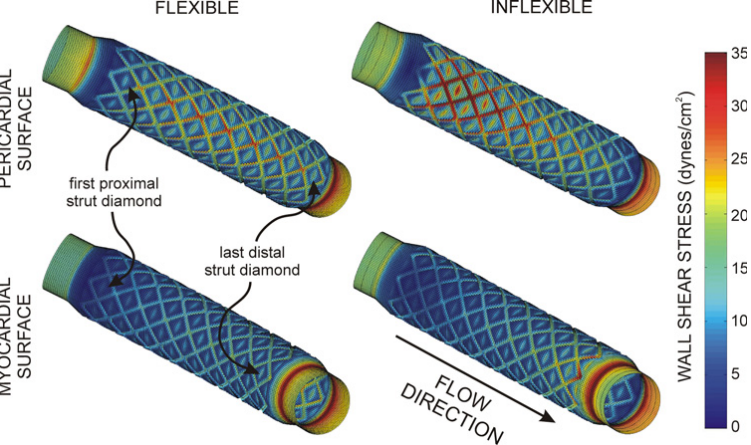
Spatial distributions of WSS along the pericardial and myocardial luminal surfaces during maximum blood flow velocity (top panel) for computational vessels implanted with 12 mm stents that conform to (flexible) or cause straightening of (inflexible) an ideal, curved coronary artery.

**Figure 6:**
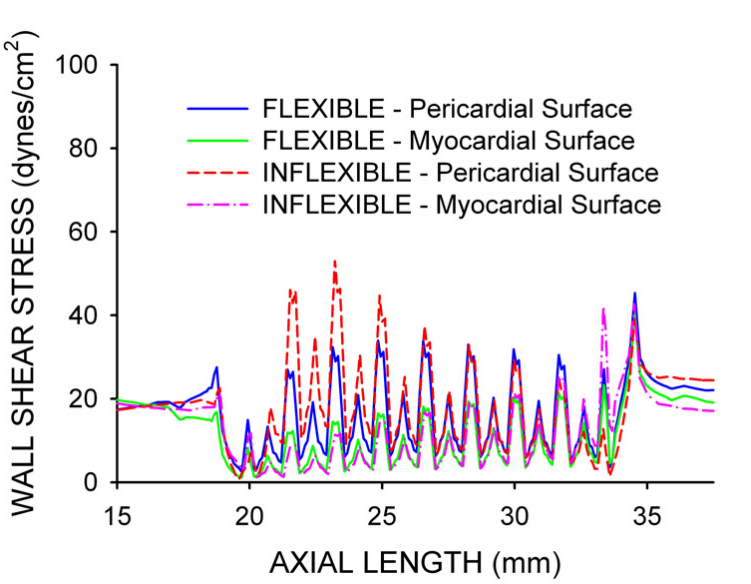
Distributions of WSS plotted as a function of normalized axial length (bottom panel) for computational vessels implanted with 12 mm stents that conform to (flexible) or cause straightening of (inflexible) an ideal, curved coronary artery.

Time-dependent alterations in the spatial distributions of WSS during the cardiac cycle are illustrated in Fig 3. Stagnation regions observed adjacent to stent struts were absent during maximum blood flow, but developed during deceleration and persisted until the onset of flow acceleration corresponding to left ventricular diastole in the coronary artery blood flow velocity waveform. Distributions of WSS were similar for both simulations. However, the presence of the inflexible stent caused a pronounced area of high WSS in the proximal portion of the stent that remained elevated throughout the cardiac cycle as compared to the flexible stent. The geometric influence of inflexible stent implantation was most pronounced during maximum blood flow velocity as illustrated by the centerline velocity profiles at several axial locations shown in Fig 4 and spatial WSS distributions presented in Fig 5. Examination of WSS as a function of normalized axial length (Fig 6) revealed that WSS was greater at the pericardial as compared to the myocardial luminal surface. This observation most likely resulted from skewing of the velocity profile. In contrast, WSS was greatest along the myocardial luminal surface at the proximal edge of the first stent strut and last distal axial strut diamonds of the inflexible stent. Increases in WSS along the pericardial luminal surface of the inflexible stent were alleviated as blood flow advanced toward the middle of the stent. Notably, distributions of WSS were more uniformly distributed along the axial length of the pericardial and myocardial luminal surfaces of the simulated coronary artery implanted with a flexible stent. There is a repeating pattern of higher WSS followed by intermittent, slightly lower WSS within the stented region of Fig 6. These localized increases in WSS are caused by the struts and the protrusion of the lumen through the openings of the stent, respectively.

**Figure 7:**
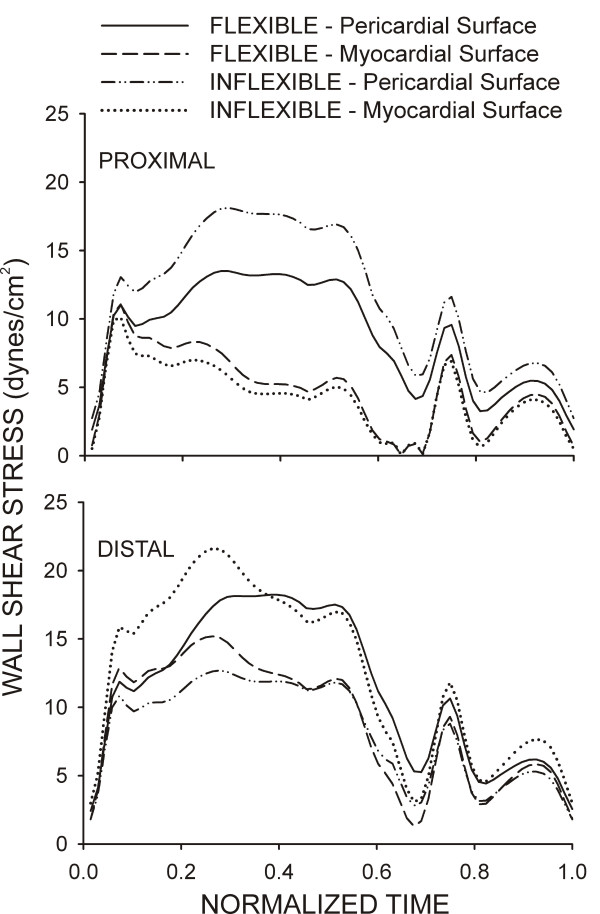
Temporal WSS distributions from the center of the first proximal and last distal repeating axial diamond along the pericardial and myocardial luminal surfaces of computational vessels implanted with 12 mm stents that conform to (flexible) or cause straightening of (inflexible) an ideal, curved coronary artery.

Temporal WSS distributions from the center of the first proximal and last distal axial diamond along the pericardial and myocardial luminal surfaces are shown in Fig 7. The TAWSS was elevated at the proximal pericardial and distal myocardial luminal surfaces of the straightened stented section as a result of the reorientation of the velocity profile as blood flow enters and leaves the region, respectively. Conversely, computational implantation of the curved stented section resulted in a gradual transition from the vessel to the stent allowing blood flow to redeveloped shortly after entering the proximal portion of the stent and restoring the characteristic modest skewing of the velocity profile toward the pericardial luminal surface resulting in elevated TAWSS along the outer as compared to the inner luminal surface. Temporal WSS distributions were spatially quantified from the first and last axial repeating stent strut diamond in the current investigation (Table 2). Similar TAWSS results in other regions of the stent may be inferred by conservation of mass and examination of Figures 3, 5 and 6 which illustrate that inflexible stent implantation subjects the proximal myocardial luminal surface to lower TAWSS because pericardial luminal WSS is elevated.

**Table 2:** Time-averaged wall shear stress (dynes/cm^2^)

	**PROXIMAL**		**DISTAL**	
**Simulation**	**Pericardial Surface**	**Myocardial Surface**	**Pericardial Surface**	**Myocardial Surface**
**FLEXIBLE**	8.91	4.88	11.5	8.93
**INFLEXIBLE**	11.7	4.26	8.28	12.6

**Figure 8:**
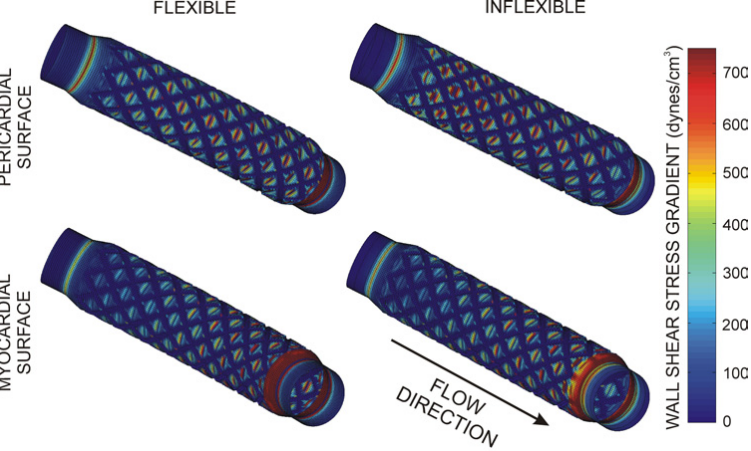
Spatial distributions of WSSG along the pericardial and myocardial luminal surfaces during maximum blood flow velocity for computational vessels implanted with 12 mm stents that conform to (flexible) or cause straightening of (inflexible) an ideal, curved coronary artery.

Spatial WSSG in flexible and inflexible stent implantation simulations are depicted in Fig 8. Redirection of the blood flow velocity profile resulting from straightening of the computational coronary artery produced prominent elevations in WSSG along the proximal pericardial and distal myocardial luminal surfaces.

## Discussion

Stents are frequently implanted in curved arterial vessels (e.g., the coronary circulation) as a treatment for critical stenoses, but most computational studies examining flow patterns through stented vessels conducted to date have used a straight cylindrical geometry. However, implantation of stents into native coronary arteries may produce regional vascular deformation because mechanical properties of the implanted stent[11,32], and changes in curvature caused by stent implantation appear to correlate with sites of restenosis[11,33]. Restenosis may occur as a consequence of changes in local fluid dynamics that occur after the stent has been implantation. Examination of blood flow patterns and WSS in theoretical vessels that more accurately reflect the *in vivo *environment may provide additional insight into this process. In the current investigation, we examined the hypothesis that the ability of a theoretical implanted stent to conform to the natural curvature of a coronary artery uniquely influences indices of WSS using 3D CFD models generated using measurements derived from native canine coronary arteries.

The current results illustrate how the blood flow velocity profile and resulting distributions of WSS are affected by changes in the primary curvature of a simulated coronary artery theoretically implanted with a stent that either conforms to or causes the straightening of this curvature. Stent conformation to the native coronary arterial curvature produced a gradual transition from the vessel to the stent. As a result, blood flow velocity redeveloped rapidly after entering the proximal portion of the stent and produced spatial distributions of WSS that were similar along the respective pericardial and myocardial luminal surfaces for the remainder of the stented region. Conversely, implantation of a stent that caused straightening of the artery resulted in large alterations in spatial distributions of WSS that were especially pronounced in the proximal and distal portions of the stent where blood flow enters and leaves the stented zone (Figures 5 and 6). The TAWSS also suggested that the proximal myocardial surface may be most susceptible to the development of NH as the lowest TAWSS occurred in this region of both the flexible and inflexible stent. When compared with our previous findings[8], the current results demonstrate that implantation of a theoretical stent that either conforms to or ignores the natural curvature of a coronary artery produces TAWSS that vary substantially from the value of 8.31 dynes/cm^2 ^observed throughout the entire stented region of a straight vessel implanted with a 12 mm stent[8]. The variations in TAWSS observed in curved stent simulations likely resulted from skewing of the local velocity profiles within the stented region.

The purpose of this investigation was to examine acute changes in distributions of WSS in curved, idealized coronary arteries after implantation of a stent that either conform to or causes straightening of the vessel with potential application to observed localized changes in neointimal hyperplasia. This acute response is important for several reasons. First, restenosis after stent implantation varies with stent geometry as demonstrated by several clinical journal articles[4,6,11]. This response occurs despite controlling for vessel injury strongly suggesting that the luminal geometry created immediately after implantation may explain the statistical differences in rates of restenosis observed in groups of patients treated with different stents. We previously demonstrated that the spatial distributions of WSS created after stent implantation strongly correlate with NH and function to temporally abolish WSS disparity[7]. In this previous study, NH quantified using histology was localized to the stented area and occurred primarily in areas of low WSS. Time-dependent increases in NH produced compensatory changes in vascular geometry and associated distributions of WSS, leading to progressive elimination of WSS disparity within the stented region. Moreover, cell migration and proliferation have been shown to reach a maximum shortly after implantation [34-36] and several early response genes are known to be activated by shear stress including c-fos, c-Jun, c-myc and egr-1[37] further suggesting that acute distributions of WSS established immediately after implantation may predispose the vessel to potentially deleterious distributions of WSS. Thus, the current results suggest that these acute distributions of WSS observed with the inflexible stent simulation may play in important role in establishing deleterious distributions of WSS and WSSG that are associated with NH and restenosis after implantation. This hypothesis is supported by the findings of Wentzel and colleagues who observed clinical evidence of restenosis in these regions[33]. Taken together, the previous and current findings further suggest that the blood flow environment created by a stent immediately after implantation may affect the temporal distribution and severity of NH by acutely influencing distributions of WSS. These data also suggest that not preserving the intrinsic curvature of an artery may cause more pronounced development of NH as compared to a stent that is flexible enough to conform to the artery of interest. However, this hypothesis remains to be fully tested *in vivo*.

Computational implantation of a rigid stent introduces straightening of the curved coronary artery and a reduction in the radius of curvature within the stented segment. This straightening causes the Dean number to increase and accounts for the observed changes in the velocity profiles depicted in Fig 4. The range of Dean numbers corresponding to the cardiac cycle shown in Fig 2 are within the viscous-dominated régime as indicated by Tada, Oshima and Yamane[20] resulting in only modest increases in the skewing of the velocity profiles toward to the pericardial luminal surface observed in the current investigation for the different simulations.

The current results should be interpreted within the constraints of several possible limitations. The objective of stent implantation into an atherogenic coronary artery is to enhance blood flow at rest and during exercise by reducing or eliminating the stenosis. In theory, the implanted stent will also create a local blood flow environment that resembles that observed after flexible stent implantation in the current investigation. Nevertheless, our observations are based upon stents implanted in idealized computational representations of healthy blood vessels, and the results may clearly be different using CFD models of vascular disease. The simulations presented here are based on inflexible and flexible stent implantation, but we also assumed a rigid-wall approximation. Previous studies have demonstrated that implantation of slotted-tube stents into canine epicardial coronary arteries reduced vessel compliance to zero within the stented region[12] suggesting that the present results may be valid within the stent. However, the results may differ from those observed clinically as several studies have also investigated the compliance mismatch introduced by stent implantation [38-41]. The curvature of the idealized coronary arteries simulated in the current investigation was based on the average curvature measured during a representative canine cardiac cycle using ultrasonic segment length transducers placed on the epicardial surface of the LAD perfusion territory and a corresponding location on the posterior surface of the heart[12]. Therefore, the distributions of WSS that occur at other points during the cardiac cycle may differ from those observed assuming a constant vascular curvature[19,42,43]. The examination of flow profiles in the proximal coronary arteries constitutes a current area of ongoing research. Although an approximate LAD coronary artery curvature was modeled in the current investigation, the physiologic curvature may result in velocity profiles that differ from those used in the current investigation. The average velocity value corresponding to the waveform used in this investigation is on the order of that measured after acute stent implantation in humans[44]. However, blood flow values vary greatly from one person to the next and may result in different distributions of WSS than those reported here. The current investigation was also conducted using a simplified outlet boundary condition and therefore does not replicate the ability of the distal vasculature to dilate in response to local metabolic needs, or reproduce the physiologic pressure observed within the stented region. The current investigation is among the first to consider acute distributions of WSS through a stented, curved coronary arterial model, but the LAD may not be symmetric *in vivo*. In fact, Myers et *al*. demonstrated that secondary geometry and curvature may be important when describing blood flow patterns in the coronary arteries[31]. Blood was assumed to be a Newtonian fluid in the present investigation. It is possible that incorporation of non-Newtonian conditions may result in distributions of shear stress slightly different than those presented here. In a recent study that utilized Newtonian and non-Newtonian properties, disparity in the resulting indices of WSS for the respective simulations was most pronounced away from the stent struts were the distributions of WSS could be considered unlikely to elicit a neointimal response[45]. Nevertheless, future studies will be necessary to examine the influence of non-Newtonian properties on distributions of WSS.

The current results suggest that implantation of a stent that conforms to, rather than causes straightening of an artery, may be beneficial from a hemodynamic perspective. However, it is currently unclear what distributions or indices of WSS will result in a deleterious mechanobiological response after stenting. This response is likely the combined influence of spatial distributions of WSS, spatial WSS gradients and time-averaged WSS. Moreover, the exact value required to elicit a mechanobiological response likely varies for different portions of the vasculature. Time-varying simulations were conducted for the current investigation and interpretation of the results was weighted in favor of the time-averaged WSS as this value considers the influence of the entire cardiac cycle as compared to the spatial distribution of WSS corresponding to peak or mean flow alone. Nonetheless, spatial distributions of WSS and spatial WSS gradients were also included in Table 1 to be consistent with our prior investigations. Future studies will be conducted to elucidate the relative importance of each of these indices in the mechanobiological response after stenting.

The potential applicability of the current simulation results to patients with coronary artery disease must also be considered in the context of the widespread use of drug-eluting stents to treat vascular stenoses. Recent reviews have suggested that drug-eluting stents may simply delay restenosis, and restenosis rates with these devices may ultimately be very similar to bare metal stents [46-49]. Moreover, this drug-eluting stent technology may not be applicable in all patients[46,47]. The current results lend further support to the hypothesis that local blood flow patterns created by stents require consideration during design in order to minimize the potential for adverse fluid dynamics implicated in the subsequent development of NH. While there are several complementary and potentially antagonistic stent design properties that must be balanced to optimize stent performance, the current results also contribute to the growing body of evidence indicating that hemodynamic alterations associated with stent implantation may be an important factor in the clinical reduction of restenosis.

The stents modeled in the current investigation are representative of the Palmaz-Schatz, NIR and, in general, slotted-tube stents. Previous CFD studies using similar geometries demonstrated that flow separation occurred adjacent to stent struts and was caused by the acceleration and deceleration of flow as it passed over the struts[17]. These struts were primarily aligned in the axial direction and therefore caused only slight deflections in the pattern of blood flow by gently redirecting the fluid in a gradual manner. This is in contrast to more drastic and complex flow deformation that likely occurs with more intricate stent designs containing a multitude of interconnected axially and circumferentially aligned struts. Geometric differences between other commercially available stents and that modeled in the current investigation primarily involve differences in the radial and axial stent properties that may substantially influence distributions of WSS. Stent design has become extremely elaborate, but results from the current simulation with a basic geometry may be applied to more intricate designs to gain insight about which regional and local geometric properties are most important in influencing flow dynamics.

The current results reveal that pronounced alterations in WSS were localized to different regions of the implanted stent as a result of the ability of the stent to conform to the curvature of the coronary artery or cause straightening of the localized segment. This observation is consistent with previous clinical and modeling studies[11,33] and suggests that TAWSS within the stent may be predictive of the location of subsequent NH after stent implantation when potentially deleterious distributions of WSS are established within the stented geometry. Importantly, the current simulation findings have yet to be validated with a chronic model of coronary artery restenosis and future *in vivo *studies will be required to confirm this hypothesis.

## Conclusion

In summary, the current results using 3D CFD modeling indicate that distributions of WSS in idealized curved coronary arteries differ substantially from those observed in linear, cylindrical representations. The findings further demonstrate that implantation of a stent that artificially straightens the normal curvature of a coronary artery introduces profound alterations in indices of WSS that are most pronounced in the proximal and distal areas of the stented region as compared to a flexible stent that conforms to the native anatomy. These results suggest that improved stent flexibility may be beneficial not only for device delivery, but also from a fluid dynamics perspective as well. Stents with geometric and mechanical properties that successfully restore distal perfusion but produce the least disruption to the native flow environment may reduce the incidence of NH and ultimately provide maximum chronic vessel patency.

## Authors' contributions

JFL planned and conducted the experiments from which the blood flow, curvature and diameter measurements used in the current investigation were obtained, created the automated geometric construction and mesh generation algorithm, formulated fluid dynamics models, conducted simulations and wrote drafts of the manuscript. LEO assisted with the formulation of fluid dynamics models, planning and completion of simulations, and helped with data analysis. DAH assisted in the design of the computational investigation, formulated fluid dynamics models, and helped with data analysis. JRK assisted in data analysis and critical revisions of manuscript. DCW also assisted in data analysis and critical revisions of the manuscript. PSP critically reviewed the design of the computational study and experiments from which the measurements used in the current investigation were obtained, analysis of results and also provided multiple critical revisions of several drafts of the manuscript, including the final submitted manuscript. All authors read and agreed to the submission of the manuscript in its current form.
